# The genome sequence of *Bacillus anthracis* strain “Bundesheer” isolated from a postal sack in the US embassy Vienna in 2001 during the Amerithrax incident in the US

**DOI:** 10.1128/mra.00215-26

**Published:** 2026-06-22

**Authors:** Maximilian F. Mayerhofer-Rochel, Helga Plicka, Florian Himmelbauer, Hans-Jörg Hellinger, Andreas Wimmer, Monika Ehling-Schulz

**Affiliations:** 1 Department of Biological Sciences and Pathobiology, Center of Pathobiology, Institute of Microbiology, University of Veterinary Medicine Vienna 27260, Vienna, Austria; 2 NBC & Environmental Protection Technology Division, Armaments and Defence Technology Agency, Vienna, Austria; Fluxus Inc., Sunnyvale, California, USA

**Keywords:** anthrax, microbiology, bioforensics, canSNP

## Abstract

In this announcement, we present the draft genome of *Bacillus anthracis* strain Bundesheer. This strain was isolated from a postal sack in Vienna in 2001. Phylogenetic analysis using canonical single-nucleotide polymorphism (canSNP) showed that it belongs to A.Br.081 and that it is highly similar to the Ames “Ancestor” strain.

## ANNOUNCEMENT

**Fig 1:**
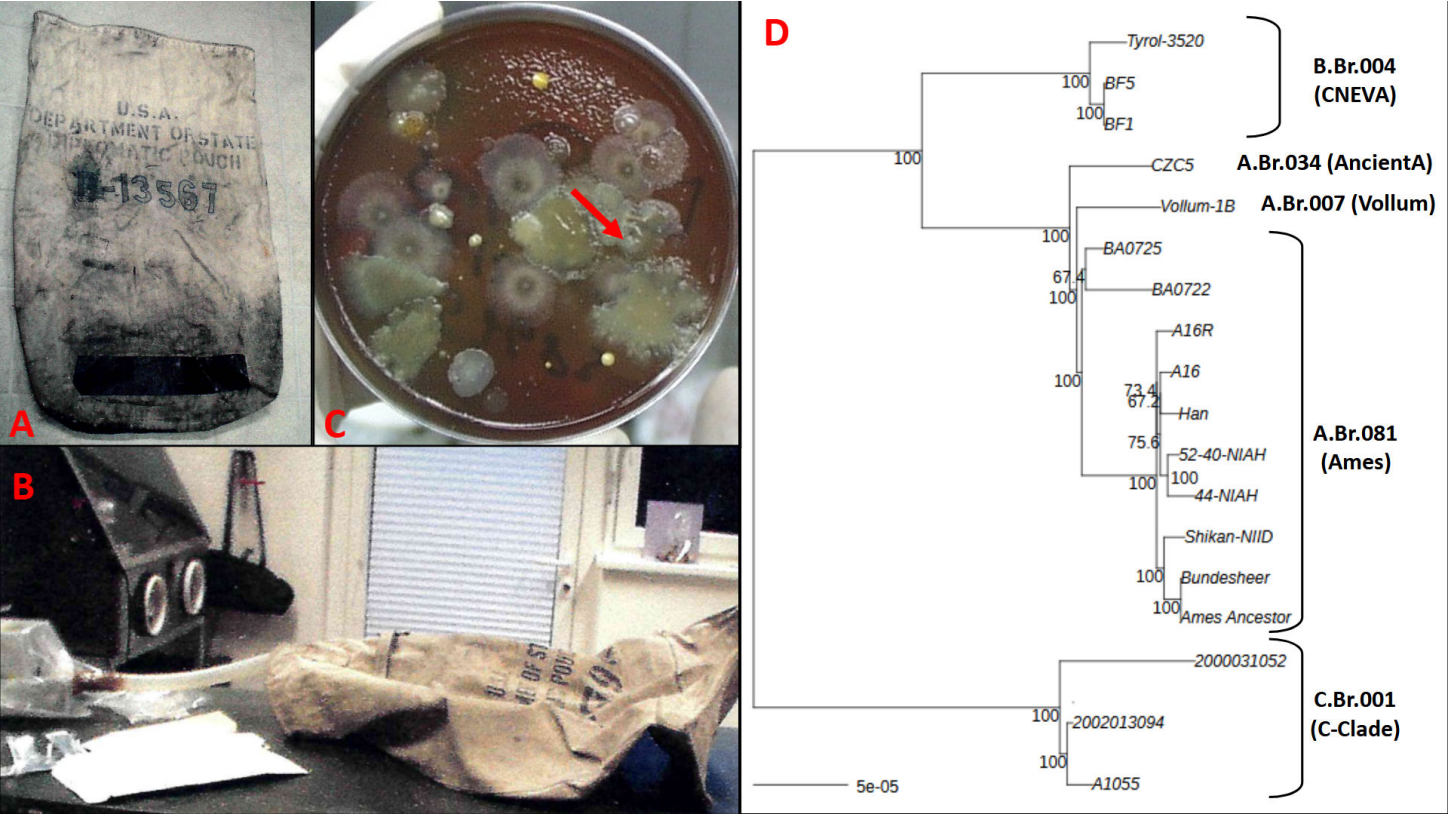
Sampling of postal sack via air sampler (**A,B**). Blood agar plate showing a mixed culture from a sampled postal sack, with an arrow highlighting the confirmed presence of *B. anthracis*. (**C**) Rooted maximum-likelihood phylogenetic tree including the isolated *B. anthracis* strain Bundesheer from a postal sack in the U.S. embassy in Austria. The tree was calculated from chromosomal SNP analysis of 3,717 SNP positions (bootstrap confidence from 500 permutations was generated, and the tree with the highest likelihood is shown) (**D**).

The Gram-positive spore-former *Bacillus anthracis* played the central role in the 2001 Amerithrax letter attacks in the United States (1). During that time, the Austrian Armed Forces were conducting an assistance operation at the U.S. embassy in Vienna. Postal sacks were sampled by collecting 500 L of air on gelatine filters (3-µm pore size, 80-mm diameter; Sartorius, USA) at 50 L/min using an air sampler (MD8; Sartorius, USA; Fig. 1). Filters were directly incubated on sheep-blood agar at 37°C for 24 h. Suspicious colonies were heat-lysed in deionized water, directly subjected to the LightCycler *B. anthracis* Detection Kit on a LightCycler 480 II system (Roche Applied Science, Switzerland), and confirmed using previously described methods (2). Colonies were stored in Microbank (Pro-Lab Diagnostics, UK), as previously described (3). Preliminary genetic analysis suggested the strain originated from natural contamination of the fabrics (4). DNA for WGS was extracted from a colony grown overnight on a Standard-I agar plate (Carl Roth, Germany) using the modified MasterPure Complete DNA/RNA Purification Kit (Lucigen, USA) with additional Ready-to-Lyse lysozyme (Lucigen, USA), as described previously (3). DNA quality and quantity were assessed using the HS Large Fragment 50-kb kit on the FragmentAnalyzer 5200 system (Agilent Technologies Inc., USA).

Whole genome sequencing was performed using the Pacbio SEQUEL I System (Pacific Biosciences, USA). Libraries were prepared according to the “Procedure & Checklist – Preparing Multiplexed Microbial Libraries Using SMRTbell Express Template Prep Kit 2.0” (Pacific Bioscience, USA) with no size selection. HMW DNA (≤2 µg) was sheared using the Megaruptor2-system (Diagenode, BEL) in 200 µL PacBio Elution Buffer with the 10-kb setting. Libraries were sequenced on SMRT Cell 1M v3 LR cells using the Sequel sequencing kit 3.0 chemistry (Pacific Biosiences, USA). The run produced 124,462 N50 (30,265 Q35) reads, with an average N50 read length of 8,259 bp (6,440 bp for Q35), quality-checked by the SMRT Link Analysis Services GUI 10.1.0.119588 software. Default parameters for all software were used except where otherwise noted. HiFi reads were used for *de novo* assembly with an expected genome size of 5.5 Mb and mapped against the reference genome *B. anthracis* str. Ames Ancestor using the SMRT Link modules Microbial Assembly and Mapping (Pacific Biosciences, USA). The assembly yielded three circular contigs (randomly rotated) corresponding to the chromosome (5,227,404 bp) and the plasmids pXO1 (181,676 bp) and pXO2 (94,832 bp), with an N50 of 5,227,404 bp, a total length of 5,503,913 bp, and a mean coverage of 35×, all determined by SMRT Link software.

QC was performed using CheckM v1.2.4 (5), BUSCO V05.02.2002 (6), and PGAP V6.8 (7) to assess completeness (98.62%), contamination (0.4%), GC content (35%), coding sequence estimate (5641), and ANI (99.97% to *B. anthracis*). The rooted maximum-likelihood tree was calculated, as previously described (3).

Phylogenetic analysis via canonical single-nucleotide polymorphism (canSNP) showed that the “Bundesheer” strain belongs to A.Br.081 (Ames) with high similarity to the Ames “Ancestor” strain (Fig. 1). The theory regarding a natural contamination of fabrics (4) can be refuted based on this finding.

## Data Availability

Raw reads and assembly data have been deposited at NCBI BioProject PRJNA1167934 (Biosample: SAMN44019342; SRA: SRS22798577, Assembly: GCF_043295875.1). The version described in this paper is the first version.
